# Magnetic Properties of GaAs/NiFe Coaxial Core-Shell Structures

**DOI:** 10.3390/ma15186262

**Published:** 2022-09-09

**Authors:** Eduard V. Monaico, Vadim Morari, Maksim Kutuzau, Veaceslav V. Ursaki, Kornelius Nielsch, Ion M. Tiginyanu

**Affiliations:** 1 National Center for Materials Study and Testing, Technical University of Moldova, 2004 Chisinau, Moldova; 2 Institute of Electronic Engineering and Nanotechnologies “D. Ghitu”, 2028 Chisinau, Moldova; 3 Institute for Metallic Materials (IMW), Leibniz Institute of Solid State and Materials Research (IFW Dresden), Helmholtzstrasse 20, 01069 Dresden, Germany; 4 Academy of Sciences of Moldova, 2001 Chisinau, Moldova

**Keywords:** nanowires, core-shell structures, anodization, electroplating, hysteresis loop, magnetic properties, coercive force, remanence ratio

## Abstract

Uniform nanogranular NiFe layers with Ni contents of 65%, 80%, and 100% have been electroplated in the potentiostatic deposition mode on both planar substrates and arrays of nanowires prepared by the anodization of GaAs substrates. The fabricated planar and coaxial core-shell ferromagnetic structures have been investigated by means of scanning electron microscopy (SEM) and vibrating sample magnetometry (VSM). To determine the perspectives for applications, a comparative analysis of magnetic properties, in terms of the saturation and remanence moment, the squareness ratio, and the coercivity, was performed for structures with different Ni contents.

## 1. Introduction

Nanowires are widely used in various applications, functionalized charge transport channels in a resonant photoeffect-transistor (RFET) [1,2] and dual piezo photo-transistors (DPPT) [3] being among them. Such kinds of devices constitute a basis for revolutionary applications in integrated optoelectronic systems, multipurpose photonic circuits, photo-triggered computing, optical processing, biomedical diagnostics, and communication.

Among other types of nanostructures, quasi-one-dimensional ferromagnetic nanostructures are of special interest for a wide range of applications, such as microelectronics, spintronics, and high-density data storage [4,5]. The properties of such structures, including those of nanowires and nanotubes, are strongly dependent on their dimensions, shape anisotropy, and selected material. Nanotubes provide advantages over nanowires, due to possibilities to vary the thickness of the tube wall in addition to the control of the length and diameter [6,7,8,9]. Even wider possibilities to control the magnetic properties are ensured by coaxial core-shell structures.

Various technologies have been developed for the production of nanoscale magnetic structures, including lithographic techniques using ion beams (IBL), focused electron beams (FEB), and two-photon technologies [8,10,11]. A drawback of these technologies is their high production cost and low throughput. A simpler, more efficient, and low-cost method has been proposed for the production of quasi-one-dimensional nanostructures, including ferromagnetic ones. It consists of the preparation of nanotemplates with a controlled arrangement of pores, and the filling of these pores with various materials. The filling of pores with magnetic materials results in the production of magnetic arrays with a desired density, aspect ratio, and composition, which ensures tunable magnetic, magneto-transport, and thermoelectric properties [6]. 

As concerns the nanotemplates for the fabrication of magnetic arrays, two types of porous structures are among the most commonly used ones, namely ion track-etched polymers and porous aluminum oxide (AAO) matrices prepared by the electrochemical oxidation of Al foils [4,6]. AAO nanotemplates are preferable ones, since they allow the easy tuning of pore diameter, length, and inter-pore distances [4]. 

The deposition of magnetic materials into nanotemplates was performed basically by atomic layer deposition (ALD) and electroplating. Electroplating was realized in various modes, such as galvanostatic mode (GM), potentiostatic mode (PM), pulsed electrodeposition (PED), and alternative current deposition (ACD) [12]. For instance, Ni and Co nanotubes have been deposited by ALD in alumina templates. Core-shell structures such as Ni-Fe_3_O_4_, as well as TiO_2_-Ni-TiO_2_, ZrO_2_-Fe_2_O_3_-ZrO_2_, ZrO_2_-Fe_3_O_4_-ZrO_2,_ SiO_2_-Fe_2_O_3_-SiO_2_, and SiO_2_-Fe_3_O_4_-SiO_2_ have been also produced [9,13,14,15,16,17]. Ni, Co, and permalloy nanowires were deposited by PM electroplating in alumina templates [18,19,20,21,22]. PED was applied for the deposition of Ni and permalloy nanowires in porous alumina or porous silicon templates [23,24,25]. Fe nanowires were deposited by ACD into alumina templates [26]. FeCo/Fe_3_O_4_ core/shell nanowires were produced by a combination of PM electroplating and ALD in alumina templates [7]. 

A limitation of producing quasi-one-dimensional ferromagnetic nanostructures with porous alumina templates is related to the fact that they are always oriented perpendicularly to the substrate surface, i.e., they are out of the substrate plane. Therefore, their magnetization is always isotropic for the in-plane orientation of the magnetic field, and they exhibit anisotropy only for the out-of-plane orientation of the magnetic field. Semiconductor nanowires provide opportunities for the fabrication of magnetic core-shell structures with different orientations with respect to the substrate surface. For instance, magnetic nanotubes have been synthesized by a combination of glancing angle and atomic layer deposition on Si nanowires, therefore ensuring a controlled inclination angle with respect to the substrate normal [13,27,28]. 

Electrochemical etching technologies have been developed for the fabrication of GaAs nanowire arrays oriented both in-plane and out-of-plane with the substrate [29,30]. The process of GM electrodeposition of Fe on GaAs substrates has been previously investigated [31,32]. The GM electrodeposition of Fe was also applied for the coating of GaAs nanowire arrays with Fe layers [31,32]. 

The goal of this paper is to deposit ferromagnetic NiFe alloy shells with different Ni content on GaAs nanowire arrays and to investigate their magnetic properties.

## 2. Materials and Methods

Used in the experiments were (111)-oriented n-type GaAs wafers with free electron concentrations of 2 × 10^18^ cm^−3^. Chips with surface of 1 × 1 cm^2^ cleaved from these wafers were sonicated in acetone for 10 min, rinsed in distilled water, and dried. Subsequently, the chips were subjected to wet chemical etching in HCl/H_2_O with a ratio of (1:3) for 2 min in order to remove the native oxide from the surface. After these preliminary preparation procedures, the samples were anodized in 1M HNO_3_ electrolyte at applied anodic potential of 4 V in an electrochemical cell with three electrodes configuration, in which the chips served as working electrode (WE). A mesh from Pt wire with the total surface of 6 cm^2^ and a saturated Ag/AgCl served as counter electrode (CE) and reference electrode (RE), respectively. An array of nanowires with the length of 45 µm oriented predominantly perpendicularly to the (111)B GaAs substrate surface was produced as a result of etching procedure. 

NiFe alloy coatings were deposited on the GaAs nanowire arrays in an electrochemical cell with three electrodes in the potentiostatic mode at applied potential of—4 V vs. saturated calomel electrode (SCE) serving as reference electrode, while the sample served as working electrode connected and fully controlled via a Biological SP-50 (Seyssinet-Pariset, France) device [33]. A Pt cage was used as counter electrode. Electrodeposition was carried out in electrolyte solutions containing NiSO_4_⋅6H_2_O (103 g⋅L^−1^), NiCl_2_⋅6H_2_O (5 g⋅L^−1^), FeSO_4_⋅7H_2_O (4.8 g⋅L^−1^), H_3_BO_3_ (25 g⋅L^−1^), and C_6_H_8_O_6_ (3 g⋅L^−1^) for NiFe layers and NiSO_4_⋅6H_2_O (100 g⋅L^−1^), H_3_BO_3_ (45 g⋅L^−1^), and C_6_H_8_O_6_ (1.5 g⋅L^−1^) for pure Ni layers with pH value of 3 for a period of time between 20 and 60 s to produce coatings with different thicknesses. The concentration of components in the solution was chosen to obtain coatings with the composition of Ni_0.65_Fe_0.35_, Ni_0.80_Fe_0.20_ (permalloy), and pure Ni layers, which was measured by energy dispersive X-ray analysis (EDX). For the purpose of comparison, films with respective compositions were also deposited on planar (111)B GaAs substrates. The morphology of the prepared samples was studied using a LEO-ZEISS Gemini 1530 (ZEISS, Jena, Germany) scanning electron microscope (SEM), equipped with EDX detector-analyzer. 

The magnetization curves of coaxial core-shell magnetic nanostructures were investigated by a vibrating sample magnetometer (VSM) from Quantum Design VersaLab™ (San Diego, CA, USA) with applied magnetic fields of up to ±3 T at room temperature. Investigations have been carried out in the in-plane configuration of the applied magnetic field for both planar substrates and GaAs nanowire arrays. In such a case, the magnetic field was applied predominantly in the radial direction for GaAs nanowires.

## 3. Results and Discussion

**Figure 1:**
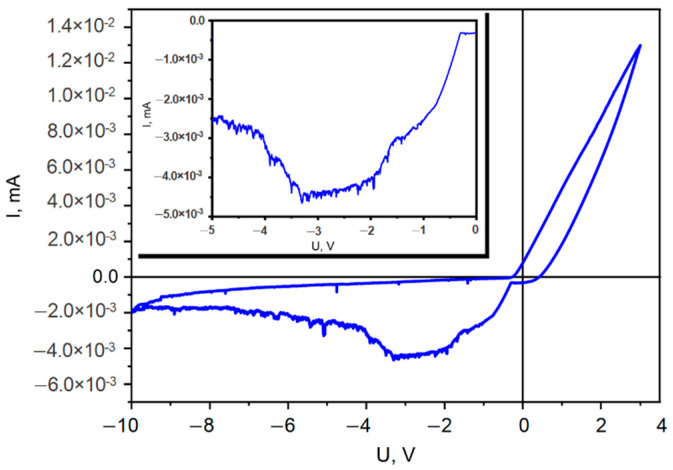
The cyclic voltammetry scan for a GaAs substrate in NiFe electrolyte. The CV scan of cathodic potential up to −5 V is presented in the inset.

Cyclic voltammetry (CV) measured with a scan rate of 10 mV⋅s^−1^ was used to study the electrochemical behavior of GaAs substrate in electrolyte containing nickel and iron salts. From CV curve (Figure 1) it can be clearly seen that processes of nickel and iron deposition take part in the potential region up to −3.3 V. At the same time, the hydrogen evolution reaction does not occur in the system, at least up to −10 V, since GaAs substrate is characterized by a moderate resistance and a reported low hydrogen evolution reaction activity [34].

The literature data of NiFe deposition disclose that the used substrate influences the electrochemical deposition process. The same electrolyte concentration was used for NiFe deposition into ion track membranes of polyethylene terephthalate, resulting in NiFe nanotubes composed from the overlapping of small nanodots at an applied potential of −1.75 V [33]. For NiFe deposition on Si semiconductor nanowires, a value of deposition cathodic potential from −1.6 V to −2 V was reported, mentioning that at −1.6 V a non-uniform deposition occurs at the ends of nanowires [35]. To note that the length of used Si nanowires was approximatively 25 µm. In our case, to polarize the whole length of the GaAs nanowires (45 µm long), a higher potential was applied. As a result, the deposition potential −4 V was chosen experimentally for uniform deposition on samples containing GaAs nanowires while at lower deposition potential, the electroplating occurs preponderantly at the bottom of nanowires.

**Figure 2:**
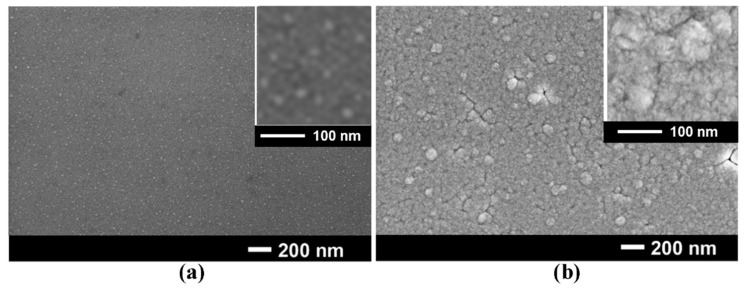
SEM images of permalloy films on GaAs substrate after 40 s (**a**) and 60 s (**b**) deposition.

The magnetic properties of coaxial GaAs/NiFe core-shell structures were compared with those of respective alloy coatings on planar GaAs wafers, for the purpose of comparison. The morphology of permalloy coatings on a (111)B GaAs wafer after 40 s and 60 s of potentiostatic deposition are shown in Figure 2. 

The coating consists of nanograins with dimensions up to 20 nm after 40 s of deposition, as deduced from the inset in Figure 2a, while the coating after 60 s of deposition represents a nanoporous layer with some randomly distributed grains with dimensions up to (70–80) nm, these having a nanoporous structure (Figure 2b). One should note that the dimensions of nanograins are less than 10 nm after 20 s of deposition, and they do not totally cover the surface of the substrate. On the other hand, conglomerates of nanorods larger than 100 nm occur after 80 s of deposition, which do not ensure a uniform thickness of the shell, and they are not suitable for the purpose of the study.

**Figure 3:**
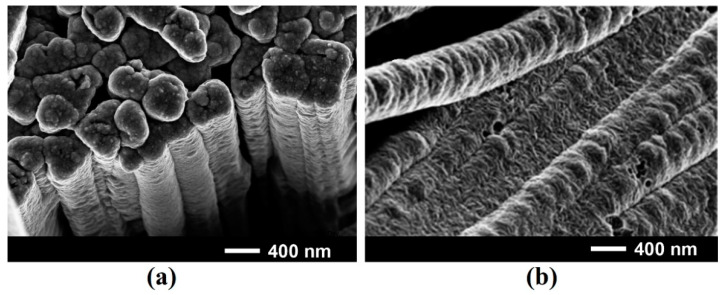
SEM images of Ni_0.65_Fe_0.35_ (**a**) and permalloy (**b**) coatings on GaAs nanowires after deposition for 60 s.

As shown in previous publications, arrays of nanowires with a 200–300 nm diameter and a 45 µm length, oriented preponderantly perpendicularly to the substrate surface preserving the initial (111)B crystallographic orientation of the substrate, are formed as a result of anodization of GaAs wafers in 1M HNO_3_ electrolyte for 15 min [29]. The morphology of both Ni_0.65_Fe_0.35_ and permalloy coatings on such arrays of GaAs nanowires have nanoporous morphology similar to that observed on planar (111)B substrates (see Figure 3).

**Figure 4:**
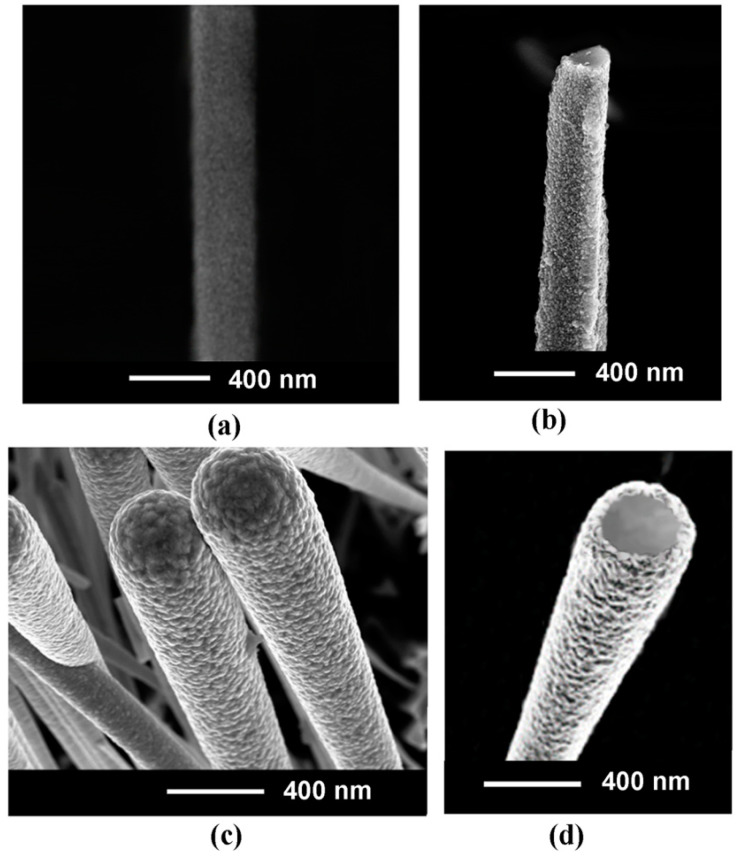
SEM images of an initial GaAs nanowire (**a**); a GaAs nanowire after Ni coating for 40 s and cutting in a plane perpendicular to the nanowire axis (**b**); a bunch of GaAs nanowires after Ni coating for 60 s (**c**); and a GaAs nanowire after Ni coating for 60 s and cutting perpendicularly to the nanowire axis (**d**).

Figure 4 shows the morphology of Ni coatings on GaAs nanowires after deposition for 40 s and 60 s as compared to the image of an initial GaAs nanowire. One can see that the distribution of nanograins is rather uniform over the surface of nanowires, and their size increases with increasing the deposition time, reaching dimensions up to around 50 nm after 60 s deposition. The thickness of the deposited Ni shell can be estimated from images of nanowires cut perpendicularly to their axis after Ni deposition.

The results of EDX analysis of the deposited alloy films summarized in Table 1 show that the compositions of films correspond within the accuracy of ±1% with those set in the electroplating solution. The deviations are determined by the accuracy of the instrument.

**Figure 5:**
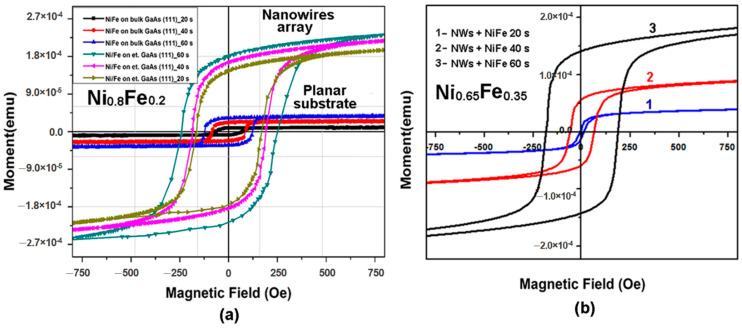
Hysteresis loops measured on permalloy-coated bulk (planar) and etched (nanowires) GaAs substrates (**a**) and Ni_0.65_Fe_0.35_-coated GaAs nanowire arrays (**b**).

Figure 5a presents hysteresis loops measured on permalloy coatings deposited in potentiostatic mode on GaAs nanowire arrays as compared with those deposited on (111)B GaAs substrates for different periods of time from 20 s to 60 s. One can see that both the magnetic moment and the coercive force are much larger for nanowire arrays than for planar substrates with similar coating thicknesses. The coatings of Ni_0.65_Fe_0.35_ alloys on GaAs nanowires (Figure 5b) are more sensitive to the deposition time as compared with permalloy coatings. However, the magnetic parameters have close values for coatings after 60 s deposition.

**Figure 6:**
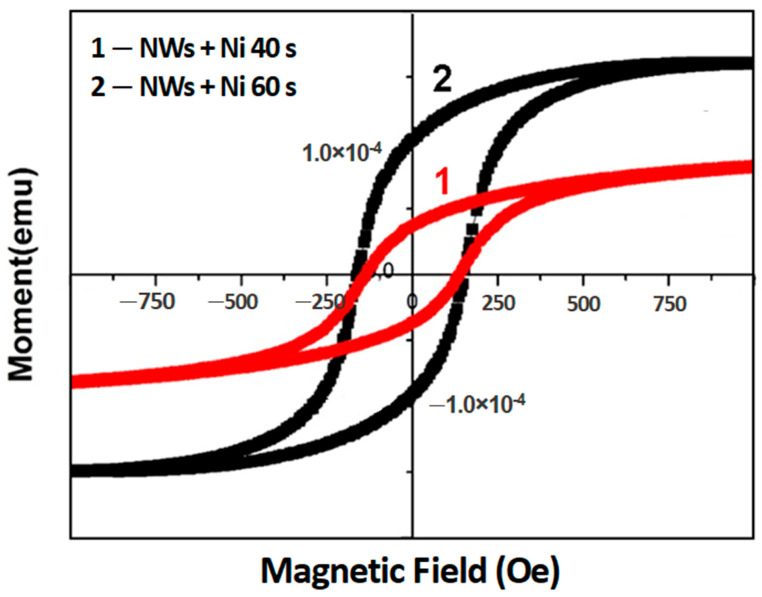
Hysteresis loops measured on GaAs/Ni core-shell arrays after Ni deposition for 40 s (curve 1) and 60 s (curve 2).

The magnetic parameters (the magnetic moment and the coercive force) of Ni coatings on GaAs nanowire arrays are also not very much different from those of Ni_0.65_Fe_0.35_ coatings after the same deposition of 60 s (Figure 6). However, the remanence ratio M_r_/M_s_ (squareness) is lower for the Ni coating as compared to the Ni_0.65_Fe_0.35_ and permalloy coatings. 

The magnetic characteristics of the investigated samples are summarized in Table 2.

The analysis of data in this table suggests that the coercive force of permalloy coatings on planar substrates is lower than that of Ni_0.65_Fe_0.35_ and Ni coatings. At the same time, the coercivity of coaxial core-shell structures is higher than that of planar structures for all the coatings. Apart from that, the coercivity of coaxial permalloy/GaAs core-shell structures is even larger than that of Ni_0.65_Fe_0.35_/GaAs and Ni/GaAs structures. The remanence ratio is around 0.67 for both planar and coaxial core-shell structures with Ni_0.65_Fe_0.35_ coatings after 60 s deposition. Nearly the same value is observed for coaxial Ni/GaAs core-shell structures, while the remanence ratio is higher (around 0.80) for permalloy/GaAs structures.

A difference in the magnetic parameters of coatings on planar substrates and on nanowires was previously reported for Fe nanoparticle films [30]. This difference was explained in terms of different crystallographic orientations of the substrates and nanowires, which can lead to different coverage with Fe nanoparticles. One needs also to take into account that the geometry of the experiment is different for films deposited on planar substrates from that of films deposited on nanowires. 

As concerns nanotube geometries, previous investigations were focused mostly on single crystalline small diameter nanotubes prepared by ALD [13,15,16,17,27]. Their magnetic properties were explained in terms of the magnetic reversal mechanisms related to the movement of different types of domain boundaries: vortex wall and transverse wall [17,27]. On the other hand, the nanotubes forming the shell in core-shell structures prepared in this paper, as well as the Fe shells electroplated in the galvanostatic deposition mode on GaAs nanowires in a previous paper [30], are nanogranular and polycrystalline in their nature. As a result, the observed magnetic behavior of polycrystalline tubular structures is influenced both by the nanograin morphology and the nanotube geometry, and the movement of different types of domain walls is additionally influenced by the nanograin boundaries [30].

## 4. Conclusions

The results of this study demonstrated that magnetic parameters of GaAs/NiFe structures increase with increasing the NiFe coating thickness. The coercivity of planar GaAs/permalloy structures is lower than that of planar GaAs/NiFe structures with a lower or higher content of Ni, while this does not hold for arrays of coaxial GaAs/NiFe core-shell structures. At the same time, the coercivity of coaxial core-shell structures is larger than that of planar structures. The obtained results pave the way for the exploration of magnetic properties of coaxial core-shell structures for enlarging the area of their applications.

## Figures and Tables

**Table 1:** Elemental composition of NiFe alloy films electroplated by potentiostatic deposition.

Alloy	Element	Weight %	Atomic %
Permalloy	Ni	82	81
	Fe	18	19
Ni_0.65_Fe_0.35_	Ni	66	65
	Fe	34	35
Ni	Ni	100	100

**Table 2:** Saturation moment, remanence, and coercive forces for electrochemically deposited NiFe and Ni coatings on planar substrates and GaAs nanowires samples for VSM measurements.

Sample Description	Deposition Mode	Saturation Moment, M_s_ (emu) × 10^−4^	Remanence, M_r_ (emu) × 10^−4^	Coercive Force, H_c_ (Oe)
Planar/Ni_0.65_Fe_0.35_	Potentiost. 20 s	2.01	1.28	107
Planar/Ni_0.65_Fe_0.35_	Potentiost. 40 s	2.27	1.46	126
Planar/Ni_0.65_Fe_0.35_	Potentiost. 60 s	2.61	1.75	153
Nanowires/Ni_0.65_Fe_0.35_	Potentiost. 20 s	0.37	0.11	16
Nanowires/Ni_0.65_Fe_0.35_	Potentiost. 40 s	1.11	0.69	84
Nanowires/Ni_0.65_Fe_0.35_	Potentiost. 60 s	1.94	1.34	172
Planar/Ni_0.8_Fe_0.2_	Potentiost. 20 s	0.10	0.09	72
Planar/Ni_0.8_Fe_0.2_	Potentiost. 40 s	0.25	0.22	85
Planar/Ni_0.8_Fe_0.2_	Potentiost. 60 s	0.36	0.32	123
Nanowires/Ni_0.8_Fe_0.2_	Potentiost. 20 s	2.05	2.60	165
Nanowires/Ni_0.8_Fe_0.2_	Potentiost. 40 s	2.25	1.75	188
Nanowires/Ni_0.8_Fe_0.2_	Potentiost. 60 s	2.45	1.98	250
Planar/Ni	Potentiost. 60 s	0.80	0.74	134
Nanowires/Ni	Potentiost. 60 s	1.55	1.05	153

## Data Availability

The data presented in this study are available on request from the corresponding authors.
